# Potential Strategies Applied by *Metschnikowia bicuspidata* to Survive the Immunity of Its Crustacean Hosts

**DOI:** 10.3390/pathogens14010095

**Published:** 2025-01-18

**Authors:** Ji Zhang, Bingyu Li, Bingnan Zuo, Xiaodong Li

**Affiliations:** 1 Key Laboratory of Zoonosis of Liaoning Province, College of Animal Science and Veterinary Medicine, Shenyang Agricultural University, Shenyang 110866, China; 2020200158@stu.syau.edu.cn (J.Z.); 2021200171@stu.syau.edu.cn (B.Z.); 2 College of Aquaculture and Life Sciences, Dalian Ocean University, Dalian 116023, China

**Keywords:** *M. bicuspidata*, *E. sinensis* hemocytes, transcriptome, qRT-PCR verification, strategies of survival

## Abstract

*Metschnikowia bicuspidata* is the specific pathogen for “milky disease” in the Chinese mitten crab (*Eriocheir sinensis*), accounting for huge losses to the industry. And yet, there is no precise study describing the pathogenesis of *M. bicuspidata*, largely hindering the development of novel control methods against its causing diseases. Here, we compared the transcriptomes of *M. bicuspidata* cells collected from a control group (cultured without *E. sinensis* hemocytes) and a treatment group (cultured with *E. sinensis* hemocytes), using RNA sequencing. Through comprehensively analyzing the differentially expressed genes (DEGs), both the most regulated ones and the ones involved in crucial enriched KEGG pathways, we found that certain processes might be required for *M. bicuspidata*’s survival under hemocyte stress. Key genes involved in oxidative phosphorylation, fatty acid metabolism, upper glycolysis, and gluconeogenesis were upregulated, and those for β-glucan unmasking, autophagy, and cell polarity were downregulated, in the treatment group. Our results suggest that *M. bicuspidata* colonizes and therefore establishes an infection in *E. sinensis* via enhancing aerobic respiration, glucose-6-phosphate accumulation, and cell-wall masking. In addition, we applied multiple means to evaluate a series of candidate reference genes and found that *PMA1* in combination with *ACT1* is the most suitable choice for accurate normalization in quantitative real-time PCR (qRT-PCR) assays. Thus, we used this combination as the reference and performed qRT-PCR verification of several DEGs. It is shown that the expression trends of these tested DEGs in qRT-PCR assays are the same as those in RNA-Seq assays. This study not only provides insights into strategies facilitating *M. bicuspidata*’s survival within *E. sinensis*, initially elucidating the pathogenesis of this yeast, but also recommends a useful molecular tool regarding qRT-PCR assays in this pathogen.

## 1. Introduction

*Metschnikowia bicuspidata*, classified within the family *Metschnikowiaceae* of the order Saccharomycetales, phylum Ascomycota, was first described by Metchnikoff in 1884 [1]. This yeast species has three varieties, namely *M. bicuspidata* var. *bicuspidata*, *M. bicuspidata* var. *californica*, and *M. bicuspidata* var. *chathamia* [2]. Globally distributed, *M. bicuspidata* is an opportunistic pathogen infecting a broad spectrum of aquatic hosts including *Daphnia* [3,4], *Artemia* [5], *Macrobrachium rosenbergii* [6], *Portunus trituberculatus* [7], salmon [8], *Exopalaemon carinicauda* [9], and *Palaemonetes sinensis* [10]. Notably, the “milky disease” of the Chinese mitten crab (*Eriocheir sinensis*) caused by this yeast was first described in 2021 [11]. It has been reported that this disease has an infection rate in adult crabs in summer at 6.65%, escalating to 30.82% after the over-winter period in high-density ponds [12]. Initially asymptomatic, infected crabs may ultimately exhibit hepatopancreas liquefaction, transforming the tissues into a milky white mixture of substances termed “milky disease” in aquaculture vernacular [11,13]. Due to the extensive domestic trade of Chinese mitten crabs, inadvertent transportation of infected individuals has led to the propagation of the disease among local river crab populations, culminating in substantial damage to the aquaculture industry [12,14]. The immune defenses of *E. sinensis* encompass humoral and cellular immunity, with hemocytes being critical to the latter. These immune cells are vested with multifunctional capabilities such as phagocytosis, encapsulation, non-self recognition, and elimination of reactive oxygen intermediates, underscoring their significance to the crab’s immune defenses [15].

The metabolic networks within yeast cells enable them to grow, develop, and adapt to a variety of environments by providing the necessary energy. When yeast cells use glucose as a carbon source for aerobic respiration, glycolysis produces pyruvate that is then transported into the mitochondria, where it is transformed into acetyl coenzyme A (acetyl-CoA), feeding into the tricarboxylic acid (TCA) cycle. The TCA cycle is a crucial provider of energy for aerobic respiration in cells and supplies a variety of intermediate metabolites for other metabolic pathways [16]. Both the β-oxidation of fatty acids and the glyoxylate cycle can take place within peroxisomes. In β-oxidation, fatty acids are used as a carbon source, and the resulting acetyl-CoA enters the glyoxylate cycle or is exported from the peroxisomes to engage in other metabolic pathways in the cell. Succinate, a product from the glyoxylate cycle, can replenish the TCA cycle or act as a precursor for amino acid synthesis, whereas intermediate metabolites like citrate and malate may be shuttled to the cytoplasm to participate in additional metabolic pathways [17]. Like in the other eukaryotes, the expression of genes involved in main energy-metabolism pathways is co-regulated via multiple global regulators including Cyc8, Cat8, Snf1, the cAMP-PKA pathway, and the hypoxia-signaling pathway in yeasts [18,19,20,21,22].

In yeasts, energy metabolism has significant effects on processes like pathogenesis and the stress response. For instance, *Candida albicans* has the ability to utilize alternative carbon sources while consuming glucose concurrently, a characteristic considered as a virulence attribute [23]. In addition, many studies have shown that mitochondrial oxidative phosphorylation (OXPHOS) in *C. albicans* is crucial for β-glucan masking, hyphal formation, biofilm development, cell wall biosynthesis, and adaptation to stress by regulating different signaling pathways [22,24]. The accumulation of certain metabolites can trigger pathways that are required for yeasts’ survival under stresses. For example, the glucose-6-phosphate (G-6-P) generated by gluconeogenesis is vital for cell growth and is essential for nucleotide metabolism, glycosylation, cell wall biosynthesis, and carbohydrate storage [25]. Moreover, during aerobic respiration, when a considerable amount of reactive oxygen species (ROS) is leaked from the respiratory chain, *Saccharomyces cerevisiae* cells might augment the pentose phosphate pathway (PPP) to produce an abundance of NADPH to maintain redox balance and deal with oxidative stresses, which is stimulated through the accumulation of G-6-P [26].

To our knowledge, there are no specific reports regarding transcriptomic analysis for studying *M. bicuspidata*’s survival strategies under the host stresses and therefore pathogenesis. Here, we performed RNA sequencing (RNA-Seq) using *M. bicuspidata* cells incubated with and without *E. sinensis* hemocytes, respectively, to analyze the metabolic changes and regulatory functions of related proteins within the yeast under external stresses. We also employed different algorithms and programs meticulously, to evaluate the expression stability of seven candidate reference genes, and used the most suitable gene combination as the reference, for our downstream quantitative real-time PCR (qRT-PCR) tests confirming the RNA-Seq results. This study provides insights into the early steps for *M. bicuspidata* to establish infection in *E. sinensis*, which will benefit the development of anti-yeast methods against “milky disease”.

## 2. Materials and Methods

### 2.1. Yeast Strain and Cell Collection

Strain LNPJ1214, isolated from an infected specimen of *E. sinensis*, was identified as *M. bicuspidata* using PCR amplification and subsequent BLAST analysis of its ITS sequence on NCBI. It was grown in YPD broth (Solarbio, Beijing, China) at 28 °C for 16 h, then centrifuged at 5000 rpm for 5 min, washed with sterile water, and finally resuspended in the modified TC-100 medium. To obtain the modified TC-100 medium, we added 100 mL of saturated sodium chloride and 1% penicillin–streptomycin solution to each liter (L) of the commercial one (Macklin, Shanghai, China) to facilitate hemocyte survival.

### 2.2. Collection of Hemocytes

Healthy male Chinese mitten crabs, with an average weight of 16 ± 0.5 g, were sourced from the southern aquaculture base, located in Jiangsu Province, of Panjin Guanghe Crab Industry Co., Ltd (Panjin, China). These crabs were subjected to a week of acclimation in a tank with 100 L water, maintained at room temperature. During this stage, they were feed using a commercial diet bi-daily, with a complete renewal of their aquatic environment every 24 h. Prior to hemolymph extraction, the crabs were sedated through icing, followed by a thorough surface sanitation using alcohol. Hemolymph was meticulously drawn with a sterile 1 mL syringe from the arthrodial membrane situated at the base of the third pereopod. Subsequently, the hemolymph was immediately mixed in a 1:1 ratio with an anticoagulant solution, as previously described [27]. This mixture was then centrifuged at 4 °C for 5 min at 3000 rpm to harvest the hemocytes. Lastly, the hemocytes were suspended in the TC-100 medium for subsequent analyses.

### 2.3. Sampling of Yeast Cells for Total RNA Isolation

For RNA-Seq, two groups were designed: the control and the experimental (treatment). In both groups, yeast cells were seeded in the modified TC-100 medium at a density of 1 × 10^8^ cells/mL and the inoculum were aliquoted and grown in 24-well microtiter plates. In total, 5.5 × 10^5^ hemocytes were seeded and co-cultured with the inoculated yeast cells for the experimental-group wells, but not for the control-group wells. The microtiter plates were subject to incubation at 28 °C for 1 h, followed by 1 min centrifugation (4 °C, 12,000 rpm) to harvest the yeast cells, which were subsequently subjected to RNA extraction.

For reference-gene evaluation, the group design and yeast-cell cultivation were conducted in the same manner as described above, except that the microtiter plates were incubated at both 8 °C and 28 °C and that the yeast cells were sampled at 1 h, 6 h, and 12 h post-incubation. In this way, we could collect yeast cells grown under more conditions, ideal for scoring the quality of candidate reference genes. Three independent experiments were performed for each group.

### 2.4. RNA-Seq Analysis

Total RNA was isolated utilizing the TRNzol Universal Reagent (TIANGEN, Beijing, China), according to the manufacturer’s instructions. The concentration and purity of the RNA were determined using a NanoDrop 2000 (Thermo Fisher Scientific, Waltham, MA, USA). Additionally, the RNA integrity was evaluated using the RNA Nano 6000 Assay Kit on the Agilent Bioanalyzer 2100 system (Agilent Technologies, Santa Clara, CA, USA). Subsequently, the NEBNext Ultra RNA Library Prep Kit for Illumina (NEB, Ipswich, MA, USA) was applied to construct sequencing libraries based on the RNA samples, according to the manufacturer’s instructions. The readiness of these libraries for sequencing was corroborated through evaluation on the Agilent Bioanalyzer 2100 system.

Each library was sequenced on an Illumina platform. The obtained clean reads were then mapped to the reference *M. bicuspidata* genome (NCBI accession number: GCA_001664035.1; *M. bicuspidata* var. *bicuspidata* NRRL YB-4993). Gene function was annotated using several databases, including KOG (clusters of orthologous groups of proteins), Pfam (protein family), Nr (NCBI non-redundant protein sequences), Swiss-Prot (a manually annotated and reviewed protein sequence database), KO (KEGG ortholog database), and GO (gene ontology). Differential gene expression analysis was conducted using the DESeq2 tool on the BMKCloud platform (https://www.biocloud.net, accessed on 11 October 2022) with |log_2_Fold Change| ≥ 1.5 and FDR < 0.01 as the threshold for filtering. In addition, other analyses including volcano plot and KEGG enrichment were performed using BMKCloud.

### 2.5. Reference Gene Evaluation

Total RNA was isolated using the method described by Li and colleagues [28]. The concentration and purity of the RNA samples were assessed with an Epoch Microplate Spectrophotometer (Agilent BioTek, Santa Clara, CA, USA). The RNA integrity was verified via gel electrophoresis. For first-strand cDNA synthesis, 1 μg of the total RNA was used, following the FastKing gDNA Dispelling RT SuperMix (TIANGEN, Beijing, China) protocol.

Candidate reference genes were selected by combining insights from the pertinent literature and our transcriptome data (the ones with stable expression levels). These genes and their descriptions are listed in Table S1. Their specific primers were designed using the Primer Premier 5.0 software and synthesized by Sangon Biotech Co., Ltd. (Shanghai, China; Table S2). Prior to qRT-PCR, regular PCR was utilized to ascertain the specificity of the primers. Each PCR system included 10 μL 2 × Taq PCR MasterMix Ⅱ (TIANGEN, Beijing, China), 0.5 μL of each primer (10 μM), 1 μL of cDNA (synthesized as described above), and 8 μL ddH2O. The amplification program was set as follows: 94 °C for 3 min; 35 cycles of 94 °C, 60 °C, and 72 °C (with each step lasting 30 s); 72 °C for 5 min. After PCR, 1% agarose gel electrophoresis was employed to verify the specificity of the candidate reference genes.

For each candidate, the qRT-PCR assays were performed on the qTOWER3/G Real-Time PCR Detection System (Analytik Jena AG, Jena, Germany) to further evaluate their stability. Each qRT-PCR system included 10 μL 2 × ChamQ Universal SYBR qPCR Master Mix (Vazyme Biotech, Nanjing, China), 0.4 μL of each primer (10 μM), 1 μL of cDNA, and 8.2 μL RNAse-free water. The amplification program was set as follows: 95 °C for 30 s; 40 cycles of 10-s 95 °C and 30 s 60 °C (along with fluorescence measurement); and capture of the fluorescence signal for melting curve analysis ramped from 60 to 95 °C. Moreover, standard curves were generated from a 4-point serial dilution of pooled cDNA (1, 1/5, 1/25, 1/125), based on the qRT-PCR results, to assess the PCR efficiency for each candidate gene, which was calculated using qTOWER3/G qPCRsoft v4.0 with the formula: $E=10^{-1/\mathrm{Slope}}-1$.

The expression stability of these candidate reference genes was evaluated using different methods: geNorm v3.5 [29], NormFinder v0.953 [30], BestKeeper [31], RefFinder [32], and delta Ct method [33]. In geNorm, the pairwise variation (V) of candidate genes was calculated to determine their stability value (M). Low M values indicate high stability. Additionally, by calculating the pairwise variation in V*_n_*/V*_n_*_+1_, the optimal number of reference genes can be determined. If V_n_/V_n+1_ is lower than 0.15, *n* is considered the optimal number of reference genes [29]. NormFinder ranks candidate reference genes based on their combination of minimal intragroup and intergroup variation, represented by the stability value (S). Low S values indicate high stability [30]. BestKeeper calculates the stability values of candidate reference genes based on the average Ct value. The stability is assessed using the correlation coefficient (R), coefficient variance (CV), and standard deviation (SD). Candidate reference genes with a lower CV ± SD and higher R are considered more stable [31]. The delta Ct method verifies the stability of the candidate reference genes by calculating the average SD of the paired genes in each sample [33]. Finally, RefFinder (http://blooge.cn/RefFinder/, accessed on 13 January 2023) is used to comprehensively assess the stability of the candidate reference genes. It combines the results from the aforementioned methods to rank the reference genes based on their stability [32].

### 2.6. qRT-PCR Verification of the RNA-Seq Results

Genes subjected to qRT-PCR verification were chosen from the differentially expressed genes (DEGs; Table S3). The steps for first-strand cDNA synthesis (from the same RNA samples used for RNA-Seq) and qRT-PCR assay were the same as those described for the candidate reference genes. The expression profiles of the tested genes were normalized using the most stable reference-gene combination. Three independent experiments were performed for the verification of each gene. The relative expression of the target genes was calculated using the 2^−ΔΔCT^ method [34].

## 3. Results

### 3.1. Evaluation of the RNA-Seq Data

To investigate the effect of *E. sinensis* immunity on the transcriptome of *M. bicuspidata*, yeast cultures incubated with or without *E. sinensis* hemocytes were collected and subjected to RNA-Seq assays: see Materials and Methods for details. The statistics of our RNA-Seq data that reveal the quality of the cDNA libraries are shown in Table S4. Libraries for all the sequenced samples were qualified for the determination of gene expression. Figure S1A indicates the distribution of gene expression in the assayed samples. The reproducibility of biological replicates was assessed via Pearson’s correlation coefficient R. As shown, all samples within the experimental (treatment) group were highly correlated with each other, while in the control group, the respective correlation of C3 with C1 and C2 was obviously low (Figure S1B). Thus, we excluded the abnormal sample C3 from the control group, during the comparison of transcriptome between these two groups.

### 3.2. Initial Classification of the DEGs

**Figure 1:**
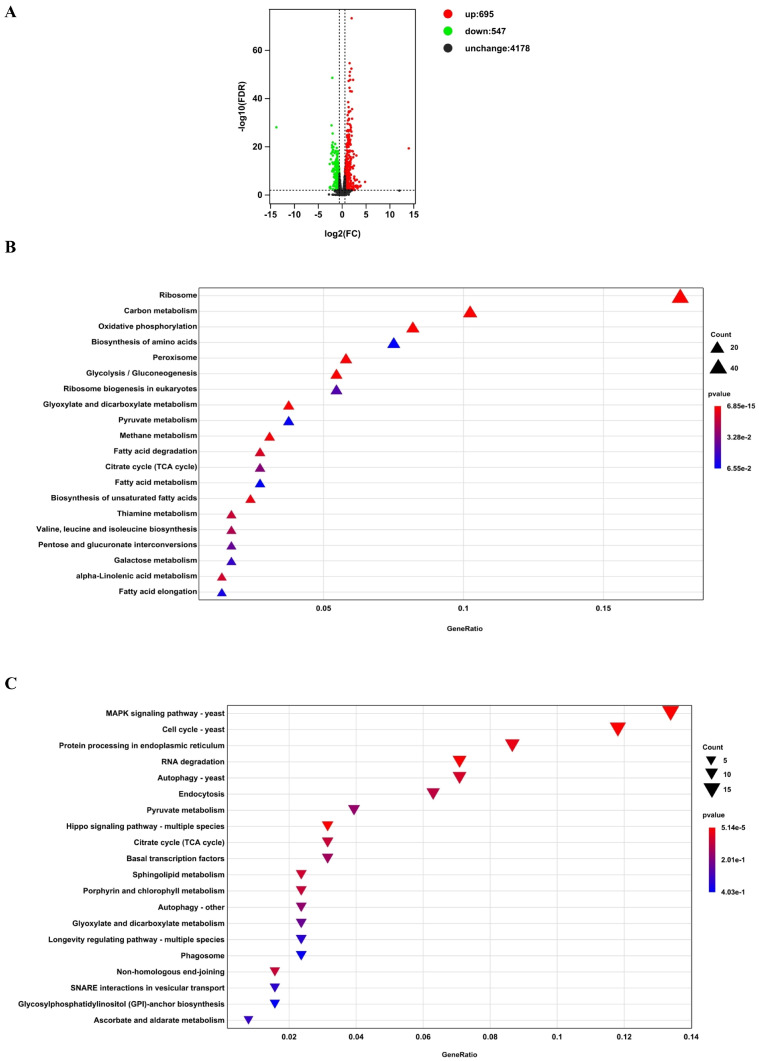
Initial analyses of DEGs according to the RNA-Seq data. (**A**) A volcano plot showing the number, distribution, and difference significance of DEGs. The gene-expression difference and its statistical significance was measured as Log_2_FC and -Log_10_FDR, respectively. Red dots, upregulated DEGs; green dots, downregulated DEGs; black dots, unchanged genes. (**B**,**C**) are charts showing KEGG pathways enriched among the up- and downregulated DEGs, respectively. The sizes of the triangles represent the number of DEGs, and the *p* values were calculated for enrichment significance.

Comparing the treatment group with the control group, a total of 1242 DEGs were obtained, of which 695 genes were upregulated and 547 genes were downregulated (Figure 1A). Using the KEGG database, 420 of the DEGs were annotated and enriched in the categories of cellular processes, environmental information processing, genetic information processing, and metabolism (Figure S2).

Out of these annotated DEGs, 293 were upregulated, with ribosome (structural ribosomal proteins) being the most affected class (Figure 1B). Taking the enriched class of ribosome biogenesis in eukaryotes (assembly of functional ribosomes) together (Figure 1B), our results suggested that a general enhancement in translation is required for *M. bicuspidata* growth during its incubation with *E. sinensis* hemocytes. Carbon metabolism was the second-place category enriched among the upregulated genes, and DEGs categorized in this pathway can also be grouped in energy-charging processes like oxidative phosphorylation (OXPHOS), or crucial metabolic pathways including the biosynthesis of amino acids, glycolysis/gluconeogenesis, citrate cycle, pyruvate metabolism, glyoxylate and dicarboxylate metabolism, and fatty acid metabolism (Figure 1B and Table S5). This is reasonable because that central carbon metabolism (CCM) not only functions in ATP producing, but also provides intermediates that are precursors for different macromolecules [35]. There were some other highly enriched upregulated pathways, peroxisome and galactose metabolism (Figure 1B and Table S5), whose functions might be important for surviving the stress of ROS and maintaining cell-wall integrity [36,37], respectively. The other pathways including methane metabolism; fatty acid degradation; biosynthesis of unsaturated fatty acids; pentose and glucuronate interconversions; valine, leucine and isoleucine biosynthesis; thiamine metabolism; fatty acid elongation; and alpha-linolenic acid metabolism seemed to be sub-categories of those crucial ones involved in CCM (Figure 1B and Table S5).

On the other hand, 127 downregulated genes were enriched in 65 metabolic pathways, in which the MAPK signaling pathway—yeast; cell cycle—yeast; protein processing in the endoplasmic reticulum; RNA degradation; autophagy; and endocytosis were enriched to a higher degree (Figure 1C and Table S5), indicating a global effect of hemocyte stress on *M. bicuspidata*. It is noteworthy that the pathways of pyruvate metabolism and citrate cycle were also concentrated, and further analysis of specific DEGs within these pathways (Figure 1B,C and Table S5), both the up- and downregulated ones, is likely to provide us more information about the mechanism(s) regarding energy metabolism, for *M. bicuspidata*’s survive in the presence of *E. sinensis* hemocytes.

### 3.3. A Glance of the Most-Affected DEGs

We investigated the top 20 up- and downregulated genes, respectively, in *M. bicuspidata* co-incubated with *E. sinensis* hemocytes, compared with those without the hemocytes. As shown in Table 1 and Table 2, we emphasized the predicted function(s) for each of the listed DEGs, according to their annotations based on the Pfam and Swiss-Prot databases.

Notably, we found that the most positively regulated (Log_2_FC = 13.96) gene, *METBIDRAFT_43176*, encodes a putative metallophosphoesterase that is also calcineurin-like (Table 1). Given the important role of calcineurin in the *S. cerevisiae* stress response [38], there is likely to be a physiological requirement of calcineurin signaling performed by the *METBIDRAFT_43176* gene product, during *M. bicuspidata*’s growth with hemocyte stress. The other upregulated genes possessed a Log_2_FC value of 2.51 to 4.79, and their functions seem to cover glycosyl hydrolyzation (*METBIDRAFT_60496* and *METBIDRAFT_40756*), glyoxylate pathway regulation (*METBIDRAFT_37387*), sugar (and other) transportation (*METBIDRAFT_227659*, *METBIDRAFT_33398*, *METBIDRAFT_12194*, and *METBIDRAFT_76577*), peroxisomal activity (*METBIDRAFT_29250*), mitochondrial function (*METBIDRAFT_46728*, *METBIDRAFT_76426*, and *METBIDRAFT_37464*), amino acid transportation (*METBIDRAFT_70890* and *METBIDRAFT_75879*), transportation by major facilitator-type transporters (*METBIDRAFT_76181* and *METBIDRAFT_79721*), and others (Table 1). Interestingly, the expression of *METBIDRAFT_33390*, whose product is a transcriptional regulator homologous with the one mediating cell adherence in *C. albicans*, was highly elevated (Table 1), providing insights into the colonization and pathogenesis of *M. bicuspidata*.

Not as informative as the upregulated DEGs, most of the downregulated ones encode hypothetical proteins (Table 2). The most negatively regulated gene, *METBIDRAFT_34094*, encodes a JmjC domain-containing protein whose homologue Epe1 can reverse gene silencing and heterochromatin in yeast (Table 2) [39]. There were six more annotated downregulated genes, whose products are homologues of the major facilitator superfamily (MFS) antiporter QDR3, suppressor of ferric uptake Sfu1, protein Pdc2, carbohydrate-binding module of glucoamylase, isocitrate lyase, and cytochrome P450 monooxygenase, respectively (Table 2).

### 3.4. Specificity and Amplification Efficiency of the Primer Pairs for Their Respective Candidate Reference Genes

**Figure 2:**
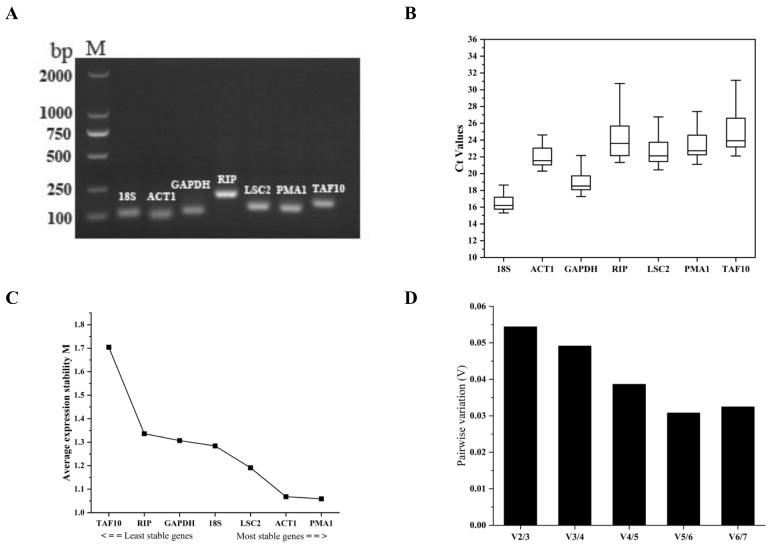
Evaluation of the candidate reference genes. (**A**) The electrophoresis result for the respective PCR amplifications of *18S*, *ACT1*, *GAPDH*, *RIP*, *LSC2*, *PMA1*, and *TAF10* (shown in Lane 2–Lane 8). Lane 1, DNA ladder. (**B**) A boxplot showing the expression levels of the seven candidate reference genes in all samples (see Materials and Methods Section 2.3) by qRT-PCR. Gene expression levels were measured as Ct values. (**C**) A chart showing the M values calculated for the candidate genes via geNorm, and the stability of these genes was sorted as indicated. (**D**) A chart showing the pairwise variation (V) values calculated for different V*_n_*/V*_n_*_+1_ combinations as indicated.

In total, we selected seven genes from the *M. bicuspidata* genome (Table 3), as candidates of the reference gene(s) for the verification of our transcriptomic results using the qRT-PCR method. The PCR products amplified using all their respective primer pairs (Table S2) showed specific bands checked via electrophoresis (Figure 2A) and all the corresponding melting curves also showed a single peak (Figure S3), indicating the good specificity of these primer pairs. In addition, the amplification efficiency of these candidate primer pairs was tested (see Materials and Methods), and they showed efficiency values from 0.90 to 1.02, with the respective correlation coefficients (R^2^) all being larger than 0.98 (Table S2). It is suggested that all the primer pairs for their respective candidate reference genes can be used for subsequent qRT-PCR experiments.

### 3.5. Analyzing the Qualifications of Candidate Reference Genes

We first analyzed the expression levels of the seven candidate reference genes in all samples (see Materials and Methods Section 2.3) by qRT-PCR (Figure 2B). Among these genes, *18S* and *TAF10* showed the highest and lowest expression levels, respectively. In detail, the average Ct values of these candidate genes ranged from 16.58 (*18S*) to 25.09 (*TAF10*). According to their SD values, we found that *18S* (16.58 ± 0.99) and *TAF10* (25.09 ± 2.61) show the lowest and highest variation in expression levels, respectively.

We then assessed the stability of these candidate genes using different systems, namely geNorm, NormFinder, BestKeeper, Delta Ct, and RefFinder, with their Ct values shown in all samples as described above. In the method geNorm, the stability is measured as an M-value and it is generally considered that a qualified reference gene should have an M-value of less than 1.5 [29]. We found that only *TAF10* possesses an M-value greater than 1.5, while that *PMA1* is the most stable gene, according to the geNorm calculation (Figure 2C and Table 3). In addition, geNorm evaluates the optimal number of reference genes by calculating the pairwise variation value (V*_n_*/V*_n_*_+1_, V-value). When the V-value is less than 0.15, the optimal number of reference genes should be *n* [29]. As shown in Figure 2D, all the V-values are less than 0.15. Therefore, two reference genes were supposed to be used for qRT-PCR verification of the transcriptomic results. The stability values of these candidate genes were calculated via NormFinder, and it was ranked as follows, *PMA1* > *ACT1* > *LSC2* > *18S* > *RIP* > *GAPDH* > *TAF10* (Table 3, small S values represent high stability). BestKeeper evaluates the expression stability of reference genes by calculating the SD and CV of the average Ct value (see Materials and Methods). When the SD value is greater than the threshold value of one, the expression of the candidate reference gene is considered unstable [31]. As shown in Table 3, among the seven candidate reference genes, only *18S* had an SD value of less than one, and *ACT1* showed a value slightly higher than one (Table 3). The Delta Ct method is dependent on calculation of the average SD for the assessed genes. The average SD of our candidate reference genes ranged from 1.059 to 1.704, with *PMA1* and *TAF10* being the most and least stable genes, respectively (Table 3).

The statistical algorithms method above created different rankings for the seven candidate reference genes. To make an accurate decision, we used the analysis tool RefFinder (see Materials and Methods Section 2.5) to evaluate the comprehensive stability of these candidate genes (Table 3). And the ranking was as follows, *PMA1* > *ACT1* > *LSC2* > *18S* > *GAPDH* > *RIP* > *TAF10*. Finally, we decided to use the combination of *PMA1* and *ACT1* as the reference for normalization of the expression of target genes that would be assessed in our upcoming qRT-PCR validation, based on the geNorm V-value result and the RefFinder ranking (Figure 2D and Table 3).

### 3.6. qRT-PCR Validation of the Transcriptomic Results

**Figure 3:**
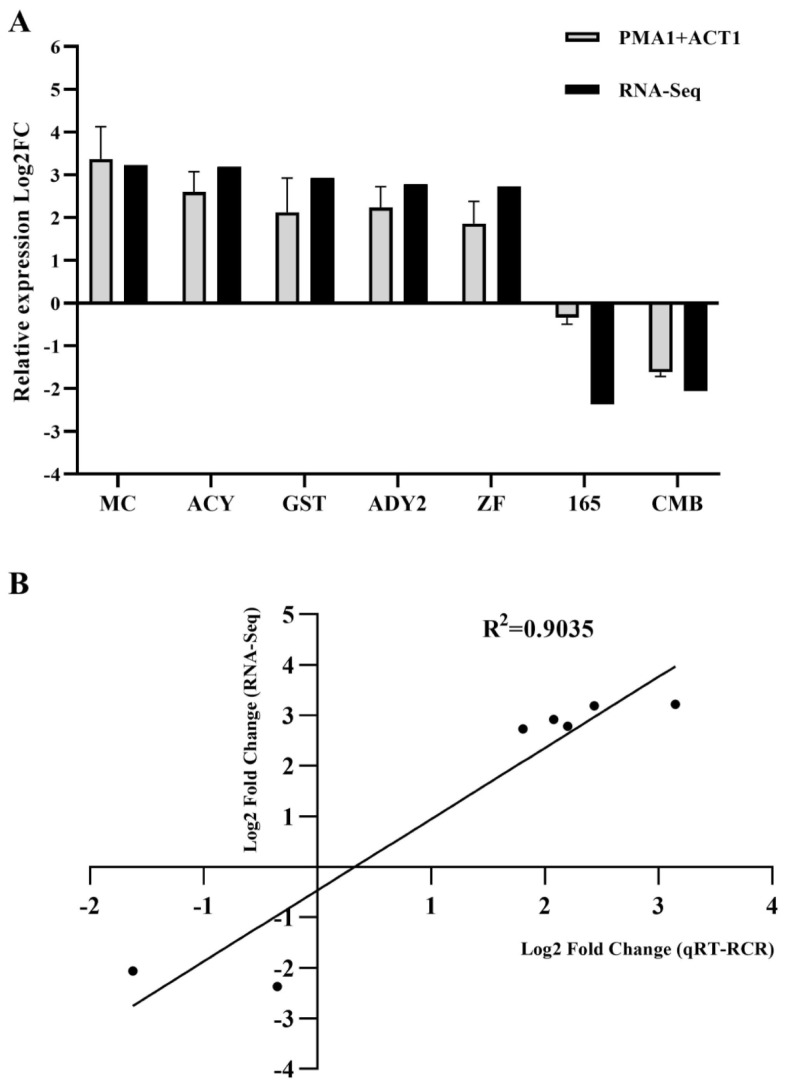
Verification of the expression patterns of certain DEGs via qRT-PCR. (**A**) A chart showing the relative expression levels of genes as indicated, in assays of qRT-PCR (gray bar) and RNA-Seq (black bar). The relative mRNA abundance (fold change, FC) for each gene in qRT-PCR was measured as described in Section 2.6 (see Materials and Methods), and the Log_2_FC values were calculated as mean ± SEM. (**B**) The fold change in the expression of these DEGs in qRT-PCR was plotted against that in RNA-Seq, and shown as a scatter chart. A trendline for these scatters was drawn and its R^2^ was calculated.

We chose several DEGs, from both the up- and downregulated groups, and subjected them to qRT-PCR validation using *PMA1* in association with *ACT1* as the reference. As shown in Figure 3A, the expression trends of all the tested DEGs observed via qRT-PCR are the same as that found in our RNA-Seq data. Moreover, the qRT-PCR results showed a comparatively high correlation with those of RNA-Seq (Figure 3B), further indicating the reliability of the RNA-Seq findings.

## 4. Discussion

*M. bicuspidata* is the pathogenic fungus causing “milky disease” in the Chinese mitten crab (*E. sinensis*), which is a serious threat to the healthy development of the river crab industry in northern China [12]. Crustaceans lack acquired immunity due to the absence of immunoglobulins and therefore rely primarily on the innate immune system, including cellular and humoral defense mechanisms. Cellular responses mediated by hemocytes are an important component of the innate immunity in *E. sinensis* [40]. Here, we investigated the reprogramming of gene expression genome-wide in *M. bicuspidata* under the *E. sinensis* hemocytes stress, to initially elucidate the manner of its pathogenesis regarding “milky disease.” We performed RNA-Seq for comparison between the control (yeast grown without hemocytes) and treatment group (yeast grown with hemocytes) in gene expression, KEGG annotation for classification of DEGs, and Pfam or Swiss-Prot annotation for functional prediction of DEGs of interest. Our data suggested that pathways involving energy metabolism, cell cycle, and signal transduction orchestrate *M. bicuspidata*’s response to the immune stress from *E. sinensis* and therefore the onset of “milky disease.”

We found that among the upregulated genes, the most enriched biological process is ribosome synthesis (including the KEGG pathways ribosome and ribosome biogenesis in eukaryotes, Figure 1B). It is reported that in *S. cerevisiae*, ribosome synthesis can be positively regulated by the target of rapamycin complex 1 (TORC1) or protein kinase A (PKA) pathway [41]. The activity of TORC1 fluctuates during the cell cycle and is inhibited during mitosis and negatively regulated by cell polarity [42]. Our data showed that the cell cycle is one of the enriched pathways among the downregulated genes (Figure 1C), which contains mitosis-related genes such as *METBIDRAFT_221572* and *METBIDRAFT_36141* (Table S5). In addition, the expression levels of genes related to stimulating cell polarity such as *METBIDRAFT_42815* and *METBIDRAFT_108669* were also significantly downregulated (Table S5). All these suggested that during co-culture with hemocytes, the TORC1 pathway of *M. bicuspidata* is activated, thereby increasing the expression of genes related to ribosome synthesis. In the case of PKA, we did not find much effective information except that the expression level of a gene (*METBIDRAFT_44477*) homologous to the gene encoding cAMP-dependent protein kinase type 2 was downregulated (Table S5), indicating that the upregulation of ribosome synthesis genes is mainly achieved through the TORC1 pathway.

Another most enriched KEGG pathway, carbon metabolism, comprises genes also belonging to certain metabolic pathways, namely OXPHOS, glycolysis/gluconeogenesis, glyoxylate and dicarboxylate metabolism, pyruvate metabolism, methane metabolism, and the citrate cycle (TCA cycle), which are enriched as well (Figure 1B and Table S5). This rough categorizing method could not provide enough information to elucidate the specific mechanisms for *M. bicuspidata*’s survival under host stresses, and yet it gave us directions for further analysis to predict the strategies of stress response in this pathogen. OXPHOS plays a crucial role in the pathogenesis of another important yeast pathogen, *C. albicans*. It has been described that OXPHOS is related to multiple aspects of *C. albicans* cell physiology that might be highly important for pathogenicity, especially its ability to transition from yeast form to hyphal form and its ability to maintain the cell wall [22,24]. Intriguingly, the expression of certain genes falling into galactose metabolism was upregulated (Figure 1B and Table S5), which indicated that maintaining cell-wall integrity and morphology is required for a quick response to crab hemocytes in *M. bicuspidata* as well [36]. However, unlike in *C. albicans*, genes important for cell polarity, filamentation, and hyphae formation (*METBIDRAFT_42815*, *METBIDRAFT_108669*, and *METBIDRAFT_47273*; Table S5) seemed to be repressed during *M. bicuspidata*’s growth with crab hemocytes, which is consistent with our findings about the yeast shape but not the hyphal shape of *M. bicuspidata* within crabs suffering “milky disease”. This suggests that *M. bicuspidata* uses a strategy different from *C. albicans* for its pathogenesis, even though OXPHOS is just as crucial.

Recently, mitochondrial function has been reported to be required for the hypoxia-induced β-glucan masking and immune evasion mediated via PKA signaling in *C. albicans* [22], meaning that OXPHOS might control *M. bicuspidata*’s crab–hemocyte response in a similar manner. β-glucan masking is a critical mechanism for fungus immune evasion and virulence and is regulated through factors including Cph1 and Crz1, in addition to hypoxia [43,44]. Notably, we found that genes (*METBIDRAFT_11928* and *METBIDRAFT_78379*) encoding Cph1 are downregulated in the presence of hemocytes (Table S5), which is likely to cause decreased β-glucan unmasking and strengthened virulence in *M. bicuspidata*. In addition, *METBIDRAFT_43176* encodes for a calcineurin-like phosphoesterase and is the most upregulated one across the genome (Table 1). Given that calcineurin signaling activates Crz1 in yeasts [38], we postulated that *M. bicuspidata* stimulates β-glucan masking through Crz1 when co-cultured with hemocytes.

**Figure 4:**
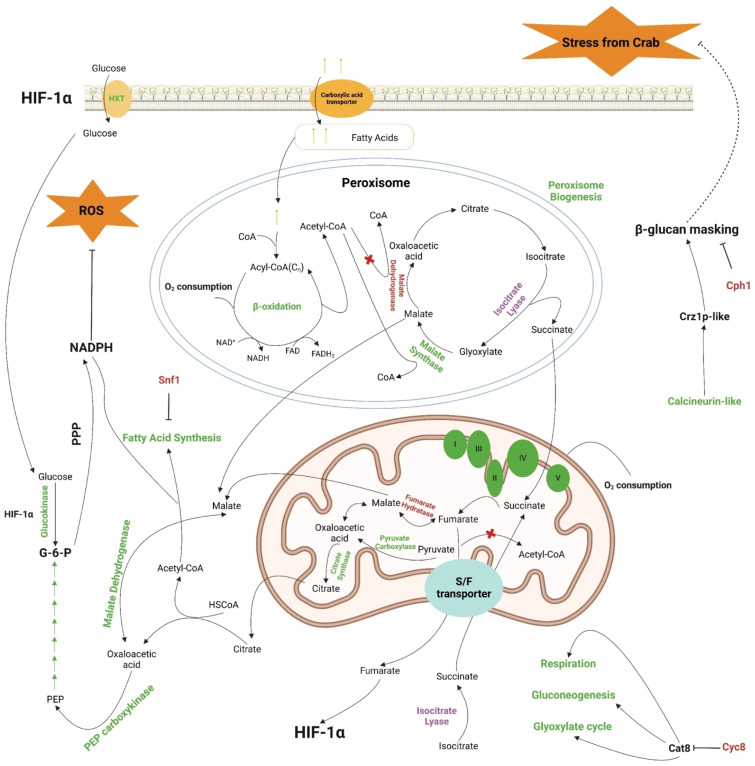
A hypothesized regulatory network based on the transcriptomic analysis and literature review. I–V, the complex I–V in the electron transport chain; words in green, genes (gene products) or pathways that are upregulated in the presence of crab hemocytes according to the RNA-Seq results; words in red, genes (gene products) or pathways that are downregulated; words in purple, genes (gene products) that can either be upregulated or downregulated; red cross, process inhibition; S/F transporter, succinate/fumarate mitochondrial transporter; HXT, hexose transporter. This cartoon was created in BioRender (https://BioRender.com/p31n089, accessed on 5 November 2024).

Interestingly, the case regarding CCM’s role in regulation of the *M. bicuspidata* crab–hemocyte response is not as straightforward. First, we noticed that genes involved in peroxisome genesis, fatty acid/carboxylic acid uptake (*METBIDRAFT_227659*), β-oxidation (fatty acid degradation), and the glyoxylate cycle were either upregulated or downregulated (Figure 1B and Figure 4 and Table 1 and Table S5). The downregulated ones are *METBIDRAFT_30113* and *METBIDRAFT_10902*, encoding peroxisomal malate dehydrogenase and a type of isocitrate lyase, respectively (Table S5 and Table 2). And yet, genes producing another type of isocitrate lyase (*METBIDRAFT_10843*) and glyoxysomal/peroxisomal malate synthase (*METBIDRAFT_92709*) were upregulated (Table S5). Along with the elevated β-oxidation and peroxisome genesis (Figure 1B and Table S5), we hypothesize that peroxisomal activity is specifically regulated to accumulate peroxisomal malate and ensure its subsequential transportation into cytosol (Figure 4) [45]. *METBIDRAFT_46728* encodes a succinate/fumarate mitochondrial transporter and was one of the most upregulated genes (Table 1). This sort of transporter specifically translocates succinate from the cytosol to the mitochondria, in exchange for fumarate [46]. This suggests that the transportation of succinate generated from the glyoxylate cycle to the TCA cycle is increased in *M. bicuspidata* challenged with crab hemocytes. Mitochondrial succinate is used as the substrate of succinate dehydrogenase (SDH), complex II in OXPHOS, generating fumarate used for the TCA cycle [47]. Our data indicated an increased expression of SDH genes except for *METBIDRAFT_39988* (Table S5), whose product is the succinate dehydrogenase iron–sulfur subunit (SDHB). The repressed SDHB expression in *M. bicuspidata* here might be a side effect of the inhibited autophagy pathway (Table S5 and Figure 1C) and does not necessarily represent dysfunction of the mitochondria, especially when the supply of iron is sufficient [48]. Moreover, we found that the mitochondrial fumarate hydratase gene (*METBIDRAFT_42115*) is downregulated, the result of which can cause the accumulation of cellular fumarate and thereby the activation of hypoxia inducible factor 1 (HIF-1) in eukaryotic cells [49]. Given that HIF-1 activates glycolysis genes producing glucose transporter and hexokinase [50], our other findings about the elevated expression of glucose-transportation genes (*METBIDRAFT_33398* and *METBIDRAFT_76577*; Table 1) and a glucose-kinase gene (*METBIDRAFT_36346*; Table S5) are reasonable due to the possibly activated HIF-1 via accumulated fumarate. However, it seemed that only the very upper glycolysis is upregulated, and genes vital to gluconeogenesis, including the ones encoding pyruvate carboxylase (*METBIDRAFT_78404*), PEP carboxykinase (*METBIDRAFT_108815*), and fructose 1,6-bisphosphatase (*METBIDRAFT_11986*), were co-activated in *M. bicuspidata* when cultured with crab hemocytes (Table S5). Along with the upregulated cytoplasmic malate dehydrogenase (*METBIDRAFT_46687*) gene and mitochondrial citrate synthase gene (*METBIDRAFT_30031*), and the downregulated mitochondrial pyruvate dehydrogenase gene (*METBIDRAFT_81264*), our data suggested a reprogrammed glycolysis/gluconeogenesis pathway that accumulates cellular G-6-P and thereby stimulates the production of NADPH through PPP, which is important for alleviating ROS stress from both the elevated OXPHOS as described above and the host hemocytes (Figure 1B and Figure 4). Interestingly, we also noticed that another pathway, fatty acid synthesis, requiring NADPH is positively regulated (Figure 1B and Table S5). It has been described that the consuming of NADPH by fatty acid synthesis might cause an increased level of cellular ROS, which could serve as a signal stimulating the regulatory network regarding energy metabolism and the oxidative stress response [51]. In accordance with this finding, we found that the Snf1-activating kinase gene (*METBIDRAFT_17838*) is decreased (Table S5), indicating repressed Snf1 activity and thus upregulated fatty acid synthesis, given that Snf1 is a known repressor of this pathway [52].

It is known that the deletion of *SNF1* results in an increased NAD(P)H concentration, enhanced Hxk2 (a hexokinase) gene transcription, and decreased mitochondrial respiration in *S. cerevisiae* grown at 1% glucose [53]. Along with its positive role in genes involved in the glyoxylate cycle and gluconeogenesis [20], we hypothesized that the specifically dysregulated carbon metabolism aiming to simulate PPP can be strongly attributed to the downregulation of *METBIDRAFT_17838* (an Snf1-activating kinase gene homologue), in *M. bicuspidata* co-cultured with crab hemocytes (Figure 4). In addition, we found that the *CYC8* gene (*METBIDRAFT_32415*) is downregulated (Table S5), which might help to explain the partially but not fully activated glyoxylate cycle, gluconeogenesis, and mitochondrial respiration (Figure 4) [19]. In other words, the obscure regulation of carbon-metabolism genes in *M. bicuspidata* challenged with crab hemocytes is likely to come from a complex effect of the simultaneously regulated Snf1-activating kinase gene and *CYC8* gene, both producing global regulators mediating energy metabolism and the stress response. Moreover, other putative regulators, such as *METBIDRAFT_37387* and *METBIDRAFT_33390* (Table 1), might contribute to glyoxylate metabolism and adherence (important for pathogenesis) in *M. bicuspidata* and are possibly crucial to its survival in hosts as well.

As described above, we mainly analyzed our RNA-Seq results through looking into the enriched KEGG pathways (Figure S2 and Figure 1B,C) and top differentially regulated genes across the whole genome (Table 1 and Table 2). It appeared that some of the most upregulated ones fit in our hypothesized regulatory network based on KEGG enrichment very well (Figure 4), suggesting the reliability of these results. Further, we verified the RNA-Seq results via subjecting certain DEGs to qRT-PCR assays. We first selected the most suitable reference-gene combination, *PMA1* and *ACT1*, after testing the specificity, amplification efficiency, and qualification (using different algorithms) of seven candidate genes (Figure 2 and Table 3). Subsequently, we used this combination as the reference and performed the qRT-PCR assay confirming the expression trends of the picked DEGs, as indicated in Figure 3A. We found that the RNA-Seq results are highly correlated with the qRT-PCR results (Figure 3B), positively supporting our findings on the stress response in *M. bicuspidata* challenged with host hemocytes.

## 5. Conclusions

In summary, this study provides insights into the possible strategies that can be used for *M. bicuspidata* to survive host immunity and to establish infection. They are β-glucan masking, NADPH production through PPP stimulation, and elevated O_2_ consumption through β-oxidation and OXPHOS (Figure 4). We certainly require many more experiments to verify our hypothesis, and studying the roles of important global regulators, namely TORC1, HIF-1, Cph1, Crz1, Snf1, Cyc8, and METBIDRAFT_37387 (glyoxylate pathway regulator), in *M. bicuspidata*’s host-stress response will be a key focus.

## Figures and Tables

**Table 1:** Most upregulated (top 20) DEGs with annotations.

Gene Name	Log_2_FC	FDR	Pfam Annotation	Swiss-Prot Annotation
*METBIDRAFT_43176*	13.96	4.54 × 10^−20^	Calcineurin-like phosphoesterase	Putative metallophosphoesterase
*METBIDRAFT_60496*	4.79	3.70 × 10^−6^	Cellulase (glycosyl hydrolase family 5)	Beta-xylosidase
*METBIDRAFT_37387*	3.85	0.000142613	GPR1/FUN34/yaaH family	Glyoxylate pathway regulator
*METBIDRAFT_227659*	3.73	0.000292482	Sugar (and other) transporter	Carboxylic acid transporter protein homolog
*METBIDRAFT_33398*	3.59	3.59 × 10^−6^	Sugar (and other) transporter	Low-affinity glucose transporter HXT3
*METBIDRAFT_12194*	3.26	0.000742583	Sugar (and other) transporter	Sugar transporter STL1
*METBIDRAFT_29250*	3.25	0.000763428	Redoxin	Putative peroxiredoxin (Fragment)
*METBIDRAFT_46728*	3.22	0.000170095	Mitochondrial carrier protein	Succinate/fumarate mitochondrial transporter
*METBIDRAFT_76426*	3.19	0.001763201	Choline/Carnitine o-acyltransferase	Putative mitochondrial carnitine O-acetyltransferase
*METBIDRAFT_70890*	3.07	4.77 × 10^−7^	Amino acid permease	General amino-acid permease GAP2
*METBIDRAFT_76181*	2.98	4.20 × 10^−17^	Major Facilitator Superfamily	Multidrug resistance protein 1
*METBIDRAFT_76577*	2.92	0.000122001	Sugar (and other) transporter	High-affinity glucose transporter HXT2
*METBIDRAFT_79721*	2.80	0.000389761	Sugar (and other) transporter	Major facilitator-type transporter ecdD
*METBIDRAFT_79129*	2.78	0.000217936	GPR1/FUN34/yaaH family	Accumulation of dyads protein 2
*METBIDRAFT_33390*	2.73	0.000286938	Zinc finger, C2H2 type	Transcriptional regulator of yeast form adherence 4
*METBIDRAFT_30436*	2.72	8.88 × 10^−7^	Phosphate transporter family	Phosphate permease PHO89
*METBIDRAFT_40756*	2.61	0.008238589	Alpha amylase, catalytic domain	Alpha-glucosidase
*METBIDRAFT_179260*	2.54	2.15 × 10^−8^	NA ^a^	NA
*METBIDRAFT_75879*	2.53	0.000199658	Amino acid permease	Proline-specific permease
*METBIDRAFT_37464*	2.51	1.44 × 10^−7^	Mitochondrial import receptor subunit or translocase	Mitochondrial import receptor subunit TOM5

^a^ NA, not available.

**Table 2:** Most downregulated (top 20) DEGs with annotations.

Gene Name	Log_2_FC	FDR	Pfam Annotation	Swiss-Prot Annotation
*METBIDRAFT_34094*	−13.78	8.25 × 10^−29^	Cupin-like domain	JmjC domain-containing protein 4
*METBIDRAFT_78276*	−2.61	1.38 × 10^−13^	Major Facilitator Superfamily	MFS antiporter QDR3
*METBIDRAFT_103598*	−2.61	0.00074696	NA ^a^	NA
*METBIDRAFT_13862*	−2.49	0.000396594	NA	NA
*METBIDRAFT_137696*	−2.44	0.001872645	NA	NA
*METBIDRAFT_165330*	−2.37	1.51 × 10^−15^	NA	NA
*METBIDRAFT_169905*	−2.29	6.12 × 10^−18^	NA	NA
*METBIDRAFT_36205*	−2.23	1.30 × 10^−29^	GATA zinc finger	Suppressor of ferric uptake 1
*METBIDRAFT_114380*	−2.12	2.10 × 10^−21^	NA	NA
*METBIDRAFT_36206*	−2.11	1.89 × 10^−20^	NA	NA
*METBIDRAFT_18009*	−2.08	1.51 × 10^−10^	DDE superfamily endonuclease	Protein PDC2
*METBIDRAFT_46171*	−2.06	2.51 × 10^−49^	Carbohydrate/starch-binding module (family 21)	NA
*METBIDRAFT_153484*	−2.04	1.44 × 10^−18^	NA	NA
*METBIDRAFT_188454*	−2.02	3.01 × 10^−14^	NA	NA
*METBIDRAFT_110843*	−2.02	6.28 × 10^−11^	NA	NA
*METBIDRAFT_30879*	−2.00	3.01 × 10^−26^	NA	NA
*METBIDRAFT_10301*	−1.98	5.37 × 10^−19^	NA	NA
*METBIDRAFT_117096*	−1.97	1.59 × 10^−22^	NA	NA
*METBIDRAFT_10902*	−1.95	4.20 × 10^−17^	Isocitrate lyase family	Isocitrate lyase
*METBIDRAFT_31334*	−1.91	1.56 × 10^−19^	Cytochrome P450	Cytochrome P450 monooxygenase

^a^ NA, not available.

**Table 3:** Expression stability ranking of the 7 candidate reference genes based on 5 algorithms.

Gene Symbol	geNorm		NormFinder		BestKeeper		Delta Ct		RefFinder	
	S ^a^	R ^a^	S	R	S	R	S	R	S	R
*18S*	1.284	4	0.654	4	0.858	1	1.284	4	2.99	4
*ACT1*	1.068	2	0.381	2	1.075	2	1.068	2	2.21	2
*GAPDH*	1.307	5	0.730	6	1.290	3	1.307	5	3.08	5
*RIP*	1.336	6	0.669	5	1.985	6	1.336	6	5.73	6
*LSC2*	1.191	3	0.552	3	1.548	5	1.191	3	2.59	3
*PMA1*	1.059	1	0.263	1	1.467	4	1.059	1	2.00	1
*TAF10*	1.704	7	1.068	7	2.124	7	1.704	7	7.00	7

^a^ S, values representing stability; R, ranking.

## Data Availability

All the raw sequencing reads from this study have been submitted to the NCBI Sequence Read Archive (SRA) under BioProject accession numbers PRJNA910793.

## Supplementary Materials

The following supporting information can be downloaded at https://www.mdpi.com/article/10.3390/pathogens14010095/s1, Figure S1: General information representing features concerning gene expression in all of the RNA-Seq samples; Figure S2: General classification of the KEGG-annotated DEGs; Figure S3: Respective melting curves for the candidate genes and tested DEGs; Table S1: Description of the candidate reference genes; Table S2: Primers used in this study; Table S3: Information of the DEGs subjected to qRT-PCR verification; Table S4: Statistics of the sequencing data; Table S5: Genes involved in the most enriched KEGG pathways.
