# Supplementary material for: Potential Strategies Applied by Metschnikowia bicuspidata to Survive the Immunity of Its Crustacean Hosts
Source: Pathogens. 2025 Jan 18;14(1):95. doi: 10.3390/pathogens14010095 (PMC11768211; doi:10.3390/pathogens14010095)
Supplement: Supplementary file 1 [file pathogens-14-00095-s001.zip › Supp. Figures-revised.pptx]

## Slide 1
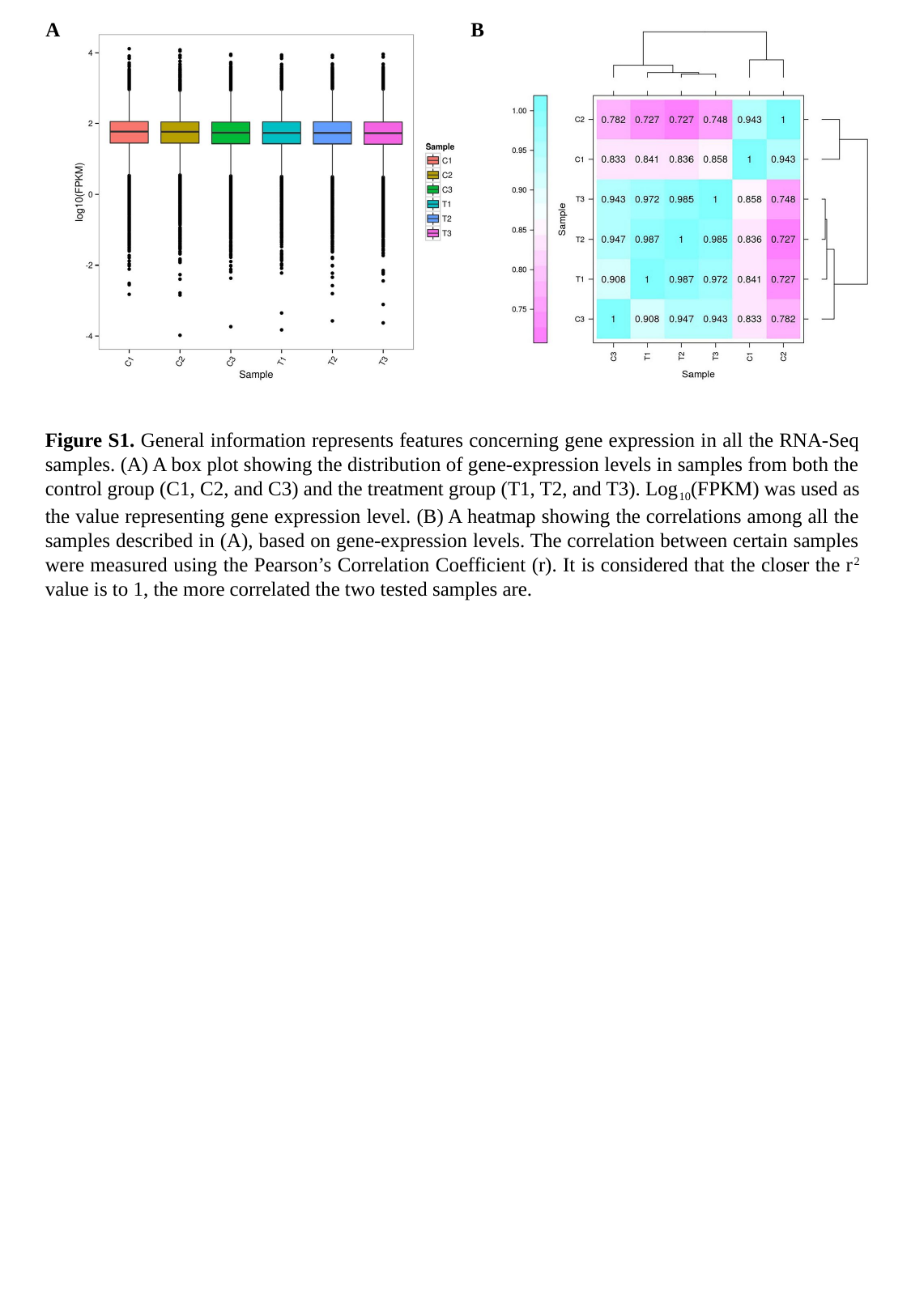

A
B
Figure S1. General information represents features concerning gene expression in all the RNA-Seq samples. (A) A box plot showing the distribution of gene-expression levels in samples from both the control group (C1, C2, and C3) and the treatment group (T1, T2, and T3). Log10(FPKM) was used as the value representing gene expression level. (B) A heatmap showing the correlations among all the samples described in (A), based on gene-expression levels. The correlation between certain samples were measured using the Pearson’s Correlation Coefficient (r). It is considered that the closer the r2 value is to 1, the more correlated the two tested samples are.

## Slide 2
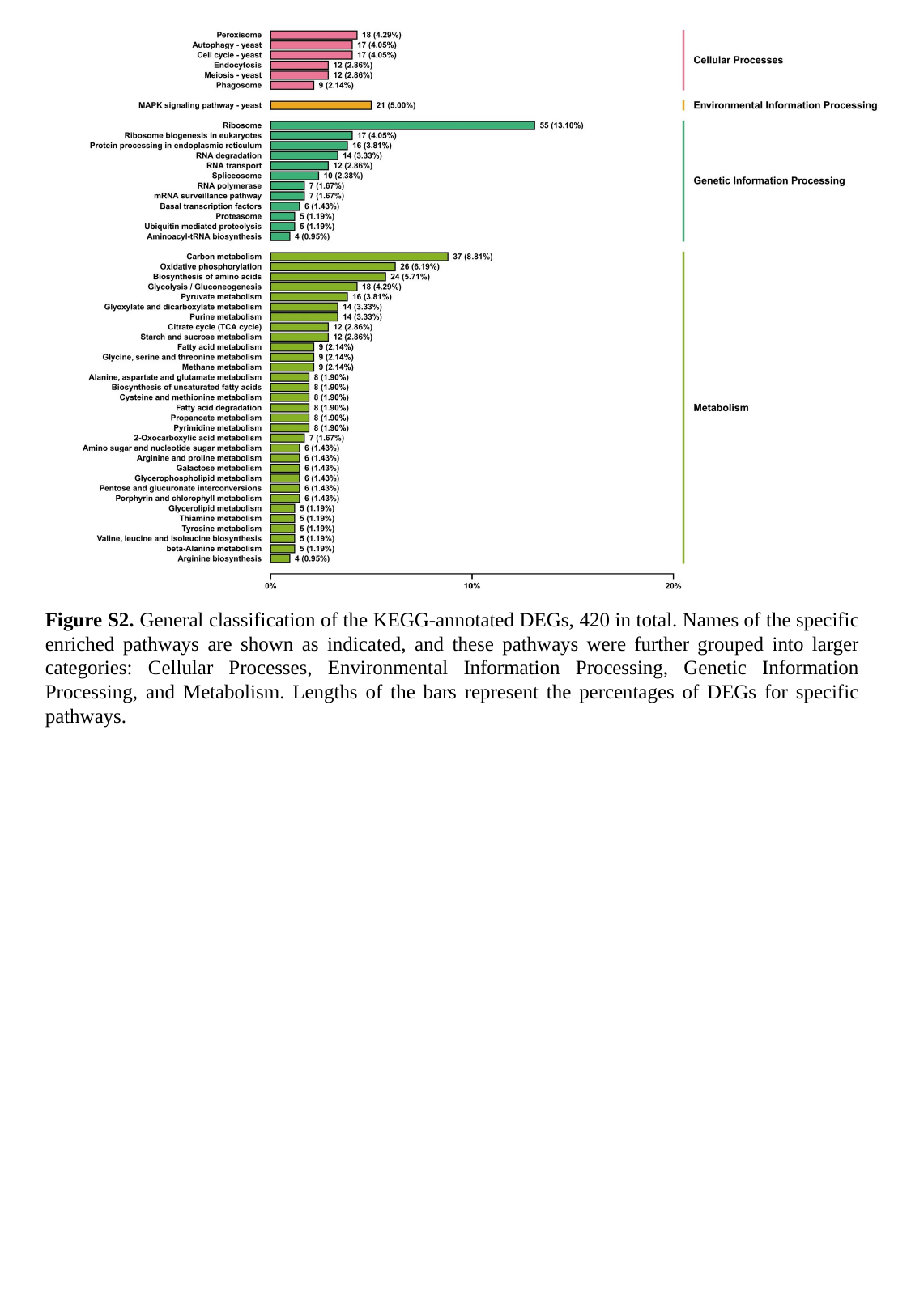

Figure S2. General classification of the KEGG-annotated DEGs, 420 in total. Names of the specific enriched pathways are shown as indicated, and these pathways were further grouped into larger categories: Cellular Processes, Environmental Information Processing, Genetic Information Processing, and Metabolism. Lengths of the bars represent the percentages of DEGs for specific pathways.

## Slide 3
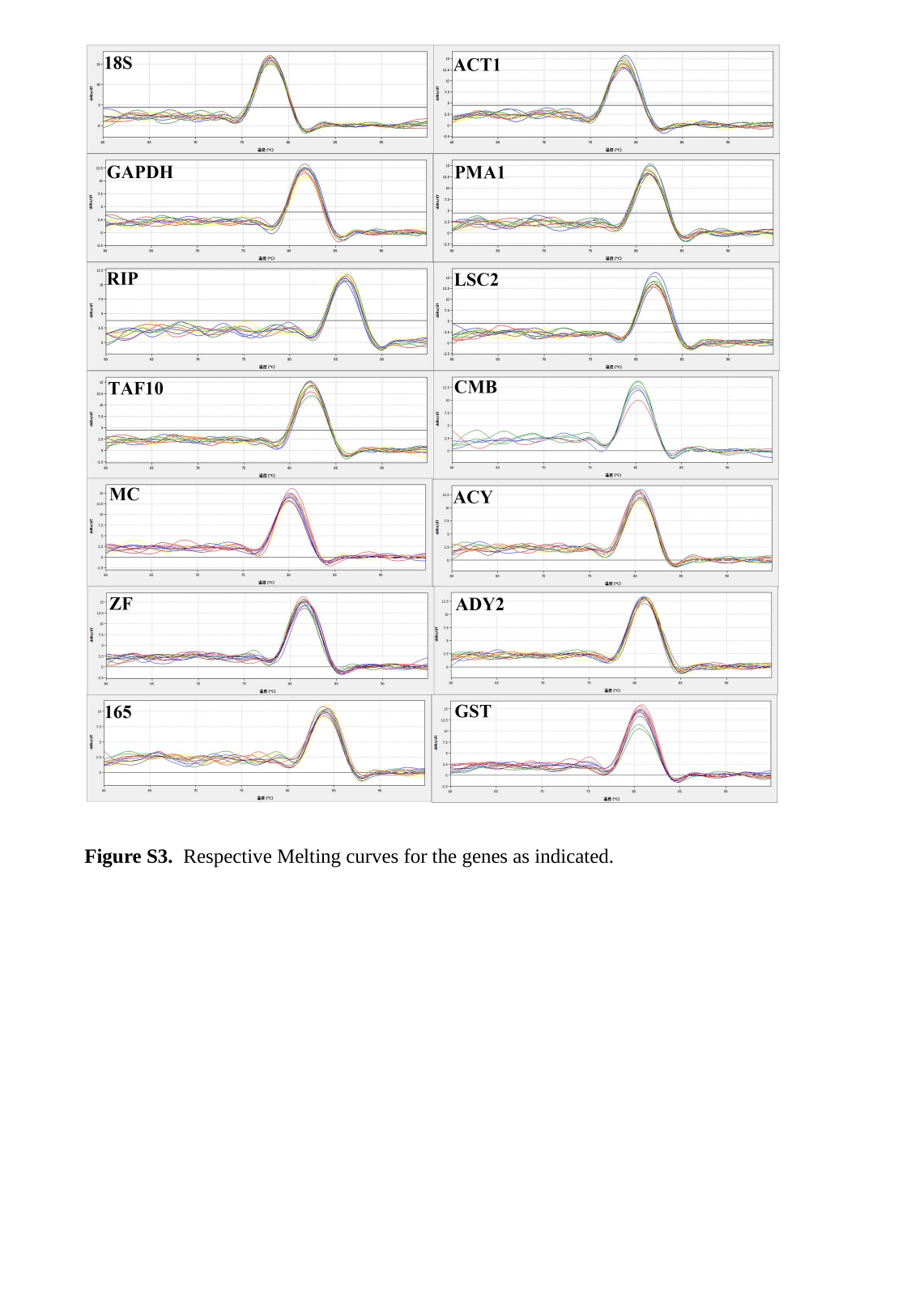

Figure S3. Respective Melting curves for the genes as indicated.
