# Supplementary material for: Potential Strategies Applied by Metschnikowia bicuspidata to Survive the Immunity of Its Crustacean Hosts
Source: Pathogens. 2025 Jan 18;14(1):95. doi: 10.3390/pathogens14010095 (PMC11768211; doi:10.3390/pathogens14010095)
Supplement: Supplementary file 1 [file pathogens-14-00095-s001.zip › Table S1.docx]

TableS1 Description of the candidate reference genes

| **Gene symbol** | **Gene product** | **Gene name** | **Function** |
| --- | --- | --- | --- |
| *18S* | 18S ribosomal RNA | X69846.1^a^ | Cytosolic small ribosomal subunit, translation |
| *GAPDH* | Glyceraldehyde-3-phosphate hydrogenase | METBIDRAFT_42262 | Oxidoreductase in glycolysis and gluconeogenesis |
| *ACT1* | Beta-actin | METBIDRAFT_223113 | Structural integrity of the cellular cytoskeleton |
| *LSC2* | Succinate-CoA ligase | METBIDRAFT_12019 | Protein present in the B-subunit cells of Succinyl CoA synthetase |
| *PMA1* | Plasma membrane ATPase 1 | METBIDRAFT_29315 | Hydrogen-exporting ATPase activity, phosphorylative  mechanism |
| *RIP* | Ubiquinol cytochrome-c reductase | METBIDRAFT_42881 | Ubiquinol-cytochrome c reductase enzyme |
| *TAF10* | transcription initiation factor IID,TAF10 subunit | METBIDRAFT_29739 | RNA pol II transcription factor activity |

^a^ The sequence of *18S* was retrieved using a NCBI accession number that is different from the locus number of *Metschnikowia bicuspidata*.
