# Supplementary material for: Potential Strategies Applied by Metschnikowia bicuspidata to Survive the Immunity of Its Crustacean Hosts
Source: Pathogens. 2025 Jan 18;14(1):95. doi: 10.3390/pathogens14010095 (PMC11768211; doi:10.3390/pathogens14010095)
Supplement: Supplementary file 1 [file pathogens-14-00095-s001.zip › Table S2.docx]

Table S2. Primers used in this study

| Gene symbol | Primer (5’-3’) | Length of PCR product (bp) | Efficiency | R^2^ |
| --- | --- | --- | --- | --- |
| *18S* | AAAGCATTTGTCAAGGACGTT  CCCAATCCCTAGTCGGCAT | 110 | 1.01 | 0.999 |
| *GAPDH* | TCTCCGTCGTTGACTTGACC  TGTAGCCCAAGACACCCTTC | 111 | 0.94 | 0.999 |
| *ACT1* | GACTCTGGTGATGGTGTTACTC  TCTCTACCAGCCAAGTTCAATC | 92 | 0.95 | 0.991 |
| *LSC2* | CTTCGTCAACATCTTCGGTGG  TCCTTGGCTTGCTTCATGTTGGTA | 135 | 1.02 | 0.998 |
| *PMA1* | TCATCGACGCCTTGAAGACCTCC  GATCTGGACAAGATGGCAATCCAC | 127 | 0.98 | 0.997 |
| *RIP* | CCATACAAGCAGCCGGACT  TAGCAGCCAACTTCACCTCGAC | 177 | 0.96 | 0.997 |
| *TAF10* | GACCTCCCAGAACTAGCGAA  TGCTAAGATCCGCTTGACC | 159 | 0.9 | 0.999 |
| *MC* | AAGAGCACGTATGCCTCTAATG  CTCGGCGTAATACCCTTGTAAA | 110 | - | - |
| *ACY* | GGAATTGGCCGGTACTTGGA  AGCACCACAGGGGAGTCATA | 113 | - | - |
| *GST* | GCTCTCACTTACTCGGTTGTCA  CGCCCACAAGAGACATGAATAC | 136 | - | - |
| *ADY2* | ATTAGCATGTTTCTACGGTGGC  TACGAGGTCAAAGCACACATGG | 96 | - | - |
| *ZF* | CGAGCTTTCACTAGGTACGAAC  GTGCCTCAAAAGTAGATCCCGT | 123 | - | - |
| *165* | TGCTCGCCACGTCTTTACC  TGTTGCAGAATTTCCCACGTT | 110 | - | - |
| *CMB* | TCCGTCACTATTCGCTACTCAG  AAGCCATTGACGAACAGGT | 149 | - | - |
